# Linking digital footprint data into longitudinal population studies

**DOI:** 10.23889/ijpds.v10i1.2946

**Published:** 2025-06-03

**Authors:** Romana Burgess, Andy Boyd, Oliver SP Davis, Louise AC Millard, Mark Mumme, Sarah Robertson, Andy Skinner, Zhuoni Xiao, Anya Skatova

**Affiliations:** 1 Population Health Sciences, Bristol Medical School, University of Bristol, UK; 2 The MRC Integrative Epidemiology Unit at the University of Bristol, Bristol, UK; 3 UK Longitudinal Linkage Collaboration, University of Bristol, UK; 4 Health Data Research UK, London, UK; 5 ALSPAC, Bristol Medical School, University of Bristol, Bristol, BS8 1QU, UK; 6 Centre for Genomic and Experimental Medicine, University of Edinburgh, Edinburgh, UK; 7 The Alan Turing Institute, UK

**Keywords:** digital footprints, longitudinal population study, ALSPAC, generation Scotland, data linkage

## Abstract

**Background:**

Linking digital footprint data into longitudinal population studies (LPS) presents an opportunity to enrich our understanding of how digitally captured behaviours relate to health traits and disease. However, this linkage introduces significant methodological challenges that require systematic exploration.

**Objectives:**

To develop a robust framework for successful digital footprint linkage into LPS, informed by discussions from a workshop from the Digital Footprints Conference 2024.

**Methods:**

We propose a structured, four-stage framework to facilitate successful linkage of digital footprint data into LPS: (1) understand participant expectations and acceptability; (2) collect and link the data; (3) evaluate properties of the data; and (4) ensure secure and ethical access for research. This framework addresses the key methodological challenges identified at each stage, discussed through the lens of two LPS case studies: the Avon Longitudinal Study of Parents and Children and Generation Scotland.

**Results:**

Key methodological challenges identified include privacy and confidentiality concerns, reliance on third-party platforms, data quality issues like missing data and measurement error. We also emphasize the role of trusted research environments and synthetic datasets in enabling secure, privacy-sensitive data sharing for research.

**Conclusions:**

While the linkage digital footprint data to LPS remains in early stages, our framework provides a methodological foundation for overcoming current challenges. Through iterative refinement of these methods there is significant potential to advance population-level insights into health and wellbeing.

## Introduction

The potential of novel data sources to inform research is always an intriguing proposition, yet progress is often hindered by reasonable concerns about data accuracy, completeness, and public trust—particularly where sensitive personal information is involved. Digital footprint data, passively collected by platforms as individuals go about their daily lives, is a case in point. While the academic community is exploring its potential value and impact [1, 2], concerns about data quality and trust remain. These data include diverse sources like supermarket transactions, smart devices, posts and interactions on social media, GPS tracking, and metrics from wearable devices. Linking these data to other datasets can provide deeper context into individual health and wellbeing, as well as improve understanding of data completeness, quality, and accuracy.

Linking digital footprint data into longitudinal population studies (LPS), which routinely monitor an individual’s health and socioeconomic status over time, can provide a more comprehensive picture of health: by connecting the detailed behavioural data from digital footprints with the depth of retrospective and prospective data available within LPS [3, 4]. However, this linkage introduces distinct methodological challenges. These issues were explored during a workshop at Digital Footprints 2024 [5], where academics working with UK LPS shared their experience of linking, curating and analysing digital footprint data.

We synthesise insights from the workshop and wider record linkage efforts to develop a methodologically grounded four-stage framework for linking digital footprint data with LPS. While the framework we propose is broadly applicable, we focus on its application in two exemplar LPS: the Avon Longitudinal Study of Parents and Children and Generation Scotland.

## Methods

### Digital Footprints Conference 2024

Digital Footprints Conference 2024 was held at the University of Bristol in May 2024 [5], drawing nearly 100 attendees from academia, industry, and government. Our linkage framework is derived from a workshop at this conference, titled “Challenges of Research with Digital Footprints in Longitudinal Population Studies,” which featured health scientists working with two prominent UK longitudinal studies—the Avon Longitudinal Study of Parents and Children^1^ and Generation Scotland^2^. The researchers presented ongoing projects using digital footprints and related data, including: fertility tracking via temperature sensors [6], health monitoring via supermarket loyalty cards [3], Twitter posts [7], mood assessment apps [8], and wearable smartwatches [9, 10]. The researchers highlighted challenges faced during these projects, informing the development of a methodological framework for successful data linkage.

### Avon Longitudinal Study of Parents and Children

The Avon Longitudinal Study of Parents and Children (ALSPAC) is an LPS based in Bristol, UK [11–13]. Pregnant women resident in Avon with expected dates of delivery between 1st April 1991 and 31st December 1992 were invited to take part in the study, leading to an initial enrolment of 14,541 mothers (referred to as Generation 0, or G0). From these pregnancies, 13,988 children were alive at 1 year of age (referred to as G1), many of which have had children of their own in recent years which have also been recruited into ALSPAC (referred to as G2, *n* = 810). The study has also expanded to include partners (*n* = 3,807 G0 partners are currently enrolled).

For over 30 years, ALSPAC has routinely collected participants’ phenotypic, environmental, genetic, and biological data through clinics, questionnaires, and linked records (e.g., GP, mental health service data, education and crime records). This comprehensive dataset is enriched by regular participant engagement. High response rates remain consistent, with 7,000+ parents completing questionnaires for G1 children during early childhood, and 4,000+ G1 participants still involved into their thirties, making ALSPAC an invaluable resource for health research. ALSPAC’s comprehensive demographic data—regularly updated and validated by participants—helps to mitigate biases by allowing researchers to control for factors like age, gender, and socioeconomic status. The longitudinal nature of the study also facilitates analyses over time, drawing associations across childhood and early adulthood.

Please note that the study website contains details of all the data that is available through a fully searchable data dictionary and variable search tool (see http://www.bristol.ac.uk/alspac/researchers/our-data/).

Recent digital footprint pilot initiatives in ALSPAC include linking participants’ supermarket loyalty cards [3], social media posts from Twitter (now X) [7], and geo-spatial data [14]. So far, around 1.5 million Tweets from 622 participants have been linked into the ALSPAC database, and 511 participants have provided consent to share their loyalty card data. Other projects have integrated data from wearables, for example: detecting smoking behaviours [9] and patterns of alcohol use [10] from smartwatches, and monitoring parent-infant interactions at home using headcams [15].

### Generation Scotland

Generation Scotland is a cohort study investigating health and wellbeing in over 36,000 participants [16]. Since its launch in 2006, data has been collected via questionnaires, biological samples, and linked records. Like ALSPAC, Generation Scotland’s comprehensive dataset spans a wide range of health, genetic, and lifestyle factors, allowing researchers to explore complex interactions between genetics, environment, and health outcomes. Its large sample size and longitudinal design strengthen analyses of trends, causality, and the impact of various factors on health over time. Plans are currently underway to increase the sample size of the study population [17].

The study has been linked to routine National Health Service (NHS) records, with the intention to extend to medical imaging and administrative datasets [17]. While digital footprint data has yet to make its debut in Generation Scotland, foundational work is underway; A recent study used a mobile app to explore loneliness in participants aged 12-15, examining emotions, social media use, and social interactions [8]. Note that while we consider digital footprint data to be passively generated independent of study participation, the loneliness app—which actively engages participants through a digital platform—is included here as an early example of integrating digital platforms into Generation Scotland, paving the way for future passive data initiatives.

## Results: The linkage framework

**Figure 1:**
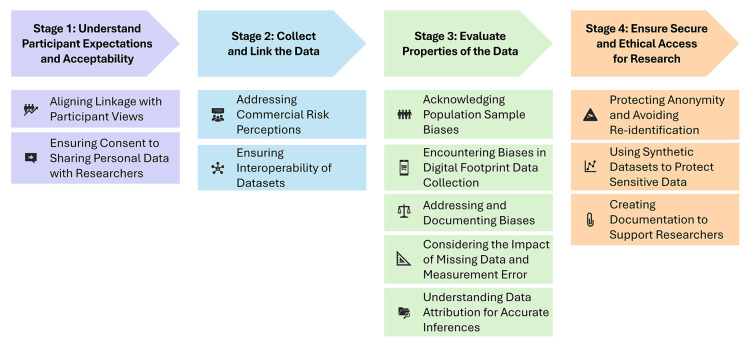
Four-stage framework for linking digital footprints data into longitudinal population studies, with associated challenges highlighted below each stage

Informed by insights from Digital Footprints 2024, we propose the following framework for successful data linkage: (1) understand participant expectations and acceptability; (2) collect and link the data; (3) evaluate properties of the data; and (4) ensure secure and ethical access for research. The framework is shown in Figure 1, with key challenges highlighted below each stage.

### Stage 1: Understand participant expectations and acceptability

The first stage relates to understanding participants’ views on the proposed linkage. Maintaining participant trust is critical for LPS, given their voluntary nature and extended durations (sometimes spanning lifetimes). These studies are grounded in transparency, ensuring participants are informed about how their data is used and retain control over it. For population data to be effectively used in research, it must have a “social licence”—public acceptance which extends beyond legal compliance and is built on trust and social benefit [18]. While LPS benefit from such a licence, incorporating potentially sensitive digital footprint data requires engaging participants early in decisions about data collection, storage, and access, addressing their expectations and concerns [19].

#### Aligning linkage with participant views

LPS research has explored how to align data linkage with participant expectations by investigating acceptability, understanding, and safeguarding participant interests in digital footprint data use [20, 21], which is key to building trust. Participant expectations can vary by data type, and linkages must be uniquely refined to incorporate these views (see Example 1).

Example 1 **Participant perceptions by data type**

An ALSPAC study found that participant perceptions of digital footprint data varied by data type [21]. Through advisory panels and focus groups, researchers found that participants perceived some data types as more sensitive (e.g., banking, GPS) than others (e.g., physical activity, sleep patterns).

This was affected by the participants’ understanding of the data and its uses, concern for third parties captured within the data, and views on the trustworthiness of the LPS.

For transaction data, recommendations to help participants feel more secure include: giving participants control over research use of their data, highlighting the data’s value, and allowing them to choose to share retrospective, future, or both types of data [22].

Public engagement is a useful tool for addressing uncertainty about data sensitivity, privacy, and security. This is exemplified by two recent exhibitions at “We the Curious” science centre in Bristol, which engaged the public in interactive discussions around tracking mental health using digital footprint data and the use of shopping data in research. In particular, these highlighted the need to break down common misconceptions about data privacy and consent, while reiterating the value of these data for public good [23, 24]. Engagement can also actively influence the direction of research (see Example 2).

Example 2 **Participant impact on research design**

The pilot of the loneliness app in Generation Scotland used participants’ opinions to shape the direction of data collection [8].

An advisory group comprised of participants from the study has influenced both technical and practical aspects of the app, including the frequency of app notifications, as well as the recruitment strategy and interface design.

#### Ensuring consent to sharing personal data with researchers

Informed consent is essential in longitudinal research to meet the study’s Common Law Duty of Confidentiality, and to uphold transparency and autonomy. Linking digital footprint data into LPS typically operates under an opt-in consent model, where participants are informed about data collection and its purpose before explicitly consenting, as exemplified by the ALSPAC supermarket loyalty card linkages [22]. Other approaches (e.g., opt-out) are at the moment unfeasible for most digital footprint data, as linkages requires IDs (e.g., social media username, loyalty card number); in contrast, opt-in consent reassures data owners that participants have willingly agreed to the use of their data.

However, while opt-in consent models reassure participants and data owners and meet some legal requirements, additional permissions are likely required from the organisations holding and controlling the data. For example, social media platforms control access via APIs, and supermarkets must usually agree to transfer loyalty card records (as they are responsible for their research use). Unlike health data, where there is precedent for research use, digital footprint data lacks a widespread expectation of sharing, making formal data-sharing agreements—ranging from simple permissions to years of negotiation—the norm for these datasets.

### Stage 2: Collect and link the data

The second stage involves collecting data from third parties and linking it with LPS records. However, digital footprint data are typically collected by a company for internal purposes (e.g., loyalty cards for marketing), leaving researchers with no control over data formats and consistency. Additionally, changes to platform algorithms, data accessibility, or privacy policies can disrupt data availability, as companies may alter how they collect information or implement stricter security measures (e.g., changes to Twitter limited API access). Such instability can affect research protocols or ethics applications.

As such, we discuss the challenges of engaging with businesses, taking into account interoperability and risk perceptions.

#### Addressing commercial risk perceptions

Businesses often have little incentive to provide data to researchers, largely due to risks of data breaches that can lead to severe penalties [25]. Competition concerns arise from sharing commercially sensitive data; for instance, if a wearables company shares data on consumer activity, competitors could benefit by tailoring their product based on this information. Companies may also worry that linking their data with LPS could expose proprietary methodologies, risking intellectual property.

These risk perceptions can lead to strict data agreements, limiting the ability to share data. Building strong partnerships with businesses can help address these concerns, ensuring that researchers and companies collaborate effectively while managing risks (see Example 3).

An alternative approach is to use data donation, where individuals voluntarily download and provide their data to researchers [26, 27]. In many cases this approach bypasses corporate complexities and gives individuals control of their data; however, it may also mean that receiving regular data updates becomes less practicable. Fortunately, technology platforms have emerged to facilitate data donation at scale. For example, the RADAR-base system—an mHealth platform leveraging data from wearables and smartphones [28]—has been used to analyse exercising behaviour during the COVID-19 pandemic [29]. Such platforms reduce the technical burden on participants and researchers, potentially making data donation a more viable and sustainable approach.

Example 3 **Building industry partnerships**

Establishing strong relationships with data providers can help secure long-term access, although this varies by provider.

For instance, partnering with a national supermarket may be more feasible due to its national focus, compared to negotiating with major international social media platforms like Meta.

In either case, navigating the corporate structure to identify a decision-maker who can grant access can be challenging, delaying access to crucial data needed for research and hindering the timeliness of studies.

#### Ensuring interoperability of datasets

A key challenge in linking data is ensuring interoperability—cohesively integrating datasets from different platforms or participants. In LPS, this can involve combining diverse digital footprint data (e.g., from an app) with cohort data (e.g., annual surveys), or linking multiple sources of digital footprint data together to create a unified dataset that is usable within the broader LPS framework (see Example 4).

Integrating these datasets presents both methodological and practical challenges, particularly in terms of the research questions they can address. While converting all digital footprint data into a single format is unrealistic due to its diversity, developing interoperable protocols and standardised methods will help researchers integrate these data with LPS more efficiently. However, we should recognise that standardisation can oversimplify data or be context specific, making the process both complicated and time-consuming.

Example 4 **Linking data from across digital sources**

In a pilot linkage, 511 participants consented to share their supermarket loyalty card data with ALSPAC. Yet, these individuals possess cards from various retailers, and are likely to vary in which is their most common place to shop.

The challenge lies in receiving these data in different formats from each retailer (e.g., variation in product categories), and finding a standardised method for formatting and integrating these disparate datasets.

### Stage 3: Evaluate properties of the data

This stage relates to data quality and understanding properties of the data that may induce bias.

#### Acknowledging population sample biases

The potential for biased samples is a concern with linked digital footprint data as it reduces the generalisability of findings. Bias can originate from several factors, including the original recruitment into the LPS and participant dropout rates. Cohort studies may retain inherent biases of their own, stemming from recruitment and data collection strategies, study location, or where certain groups are more likely to drop out than others (see Example 5).

Example 5 **Biases in cohort participants**

For instance, recent ALSPAC participants are more likely to have higher socio-economic position, better health outcomes and reduced rates of behaviours associated with poor health than the baseline enrolled participants [30].

Generation Scotland has faced similar challenges with biases in recruitment and retention, with individuals in the study being older and less socially deprived compared to the Scottish population [17].

Sociodemographic disparities raise concerns about how well findings can be extrapolated to more diverse or disadvantaged populations. Generation Scotland are addressing this by expanding geographic coverage and sociodemographic diversity among participants, ensuring the study is well-equipped to manage population biases when digital footprint data is introduced.

#### Encountering biases in digital footprint data collection

Biases can arise from the collection of digital footprint data, as individuals who engage with digital platforms and are willing to share their data may differ systematically from those who do not. Addressing digital exclusion is crucial to avoid inadvertently excluding certain groups, for example, by making assumptions about the availability, access, and affordability of digital devices (e.g., smart phones with data plans). This is both a moral and technical issue, as it leads to missing data and potential biases.

Digital footprints linkage may also face low response rates due to the sensitive and potentially identifying nature of these data (see Example 6), highlighting challenges in obtaining consent. More research is needed to understand barriers to participation, such as a lack of understanding of the research relevance, privacy concerns, or fear of study overreach.

Example 6 **Low response rates to digital footprint campaigns**

In ALSPAC, the X/Twitter campaign collected data for 18% of respondents (4261 reported having an account, 768 provided a valid public username) [7], although we know that around 43% of the UK population have an account [31].

Similarly, the supermarket loyalty cards campaign collected data for around 19% of respondents (2744 reported they owned a card, 511 agreed to linkage in a pilot exercise) [3], while around 81% of UK consumers are loyalty program members [32].

#### Assessing and documenting biases

It is therefore critical to document the underlying sample and reasons why participants are excluded from the linkage sample (e.g., attrition, active dissent, failure to respond, withdrawal, death, or departure from the UK) in a ‘consort’ style assessment. Researchers can then test linkage quality and assess if some groups are more likely to be excluded than others, as linkage can discriminate against certain socio-economic or demographic groups [33]. However, LPS have an advantage in this scenario: comprehensive baseline demographic data means researchers can identify and adjust for underrepresented groups (e.g., through inverse probability weighting).

#### Considering the impact of missing data and measurement error

Digital footprint data—like any other—is prone to missing values and measurement error (see Example 7). Issues arise from temporal gaps (e.g., user inactivity) or user behaviour (e.g., device sharing). Imputation techniques can be employed to approximate missing values, but these can lead to biased estimates depending on the specific approach used and mechanism underlying the missing data [34].

Example 7 **Biases from missing data**

In a project using wearables to track fertility [6], missing data arose from how women use the device, as they are instructed to use the device during only a portion of their menstrual cycle (after menstruation and until ovulation is predicted by the device).

This measurement error is challenging, as important temperature variability is likely to occur in the missing portions, and there are likely systematic differences in the amount (and timing) of missing data across users which may induce bias in analyses using these data.

#### Understanding data attribution for accurate inferences

Data attribution is crucial for ensuring data is “research ready” [35], using the source and provenance of data to assess accuracy, completeness, and context. However, attribution is challenging with standalone digital footprint data, as systematic missingness of data makes it difficult to accurately assess data reliability. This is a key challenge, as misattribution can lead to flawed inferences (see Example 8). LPS can help as researchers can cross-validate digital footprint data with other sources to bolster their understanding of the context.

Example 8 **Attributing individual consumption Data**

Supermarket loyalty cards may be owned by an individual, but they can capture purchases made for multiple people, making it difficult to attribute data to an individual.

Household composition, like families versus shared housing, further complicates this, as assumptions based on household data may lead to incorrect inferences about individual behaviours. Additionally, purchase does not equal consumption; buying cigarettes does not mean that the person smokes them.

In an LPS, this is addressed by using linked administrative records to infer household composition, improving the accuracy of inferences.

### Stage 4: Ensure secure and ethical access for research

The final stage involves accessing the linked data for research. A systematic review identified five key characteristics of research-ready administrative data: (1) *accessible*, with appropriate permissions and access procedures; (2) *broad*, meaning comprehensive, flexible, and linkable; (3) *curated,* with standardised, quality-checked, and up-to-date data; (4) *documented*, with information on data attributes; and (5) *enhanced*, making the data usable and meaningful for research [35]. These points also apply to digital footprint data.

#### Protecting anonymity and avoiding re-identification

Establishing robust infrastructure for data linkage, curation, and access is crucial to ensure data is research ready. Trusted research environments (TREs) are the gold standard, offering a secure interface for researchers to analyse curated data remotely within a robust governance framework. The UK Longitudinal Linkage Collaboration (UK LLC) serves as the national TRE for the UK longitudinal research community, providing record linkage services to ALSPAC, Generation Scotland, and other LPS and biobanks [36].

While TREs are well-suited for LPS data, curating digital footprint data presents challenges in safeguarding participant anonymity. Many digital footprint data are potentially disclosive: the details of payment transactions, for example, can reveal personal preferences or behaviours, while the “public” setting on social media posts means that users can be easily identified from a simple web search. To mitigate these risks, LPS data managers and TREs undertake disclosure risk assessments—such as the UK Anonymisation Networks’ ‘Anonymisation Decision Making Framework’ [37]. These frameworks guide how raw linked data should be transformed and de-identified prior to use by researchers (see Example 9), ensuring compliance with privacy laws and maintaining participant trust.

Note that while disclosure risks exist for many data types, digital footprint data pose unique challenges due to their public accessibility and unstructured nature, increasing re-identification risks. These data may contain free text and images (e.g., from social media) that require extensive disclosure controls, such as natural language processing techniques to extract structured data. While TREs provide secure access and minimise intentional misuse, they cannot fully prevent accidental recognition. Therefore, additional approaches like disclosure risk assessments, or providing synthetic data with reduced safeguards (e.g., for training purposes or feasibility assessments) may further mitigate risks.

Example 9 **Transforming identifiable data**

In a study using Twitter/X data, ALSPAC data managers derived sentiment analysis scores from participants’ raw tweets, providing de-identified scores to the researchers rather than the tweets themselves [7, 38].

This approach exemplifies how transformed outputs can be used to maintain participant confidentiality while still deriving valuable insights.

#### Using synthetic datasets to protect sensitive data

A key feature of research-ready data is accessibility: it must be easy to find and access, with appropriate permissions in place. Ethical compliance is essential, by implementing stringent access policies and security accreditations. For instance, ALSPAC requires researchers to apply for data access—undergoing ethical review—and researchers only receive the relevant data for their research. However, access to digital footprints data is limited by data sensitivity.

Synthetic datasets offer a solution, aiming to provide researchers with usable data which mimics original data patterns while safeguarding individual identities. These data are used to develop and refine analytical pipelines, allowing researchers to test models without exposing sensitive information. After developing the models, real data is used for final validation.

However, synthetic data presents a trade-off between privacy and utility. If derived from real data, there is a risk of inadvertently leaking sensitive information [39]. Alternatively, researchers can generate entirely artificial datasets or use publicly available data, which can be synthetically linked to the LPS, to enhance the dataset without compromising privacy. In either case, this may limit analytical value (e.g., if the data is oversimplified).

#### Creating documentation to support researchers

Developing comprehensive documentation for digital footprint data is both necessary—given the novelty of the data and the need for reproducible methods—and challenging, due to the involvement of third parties, and the sensitivity and complexity of the data. Early engagement with industry partners and multi-disciplinary experts (e.g., data scientists, statisticians, IT personnel) is vital to ensure that adequate metadata is collected to support datasets and their interpretation.

While there has been a drive by journals and research community to deposit datasets openly, this conflicts with confidentiality and legal requirements of digital footprint research. LPS or TREs can provide a stable means for others to access data for replication, ensuring both scientific rigor and participant protection.

## Conclusion

Linking digital footprint data into LPS presents an opportunity to enhance our understanding of physical and mental health patterns and outcomes, and socioeconomic factors and behaviours over time. The potential to combine detailed digital data with long-term historical records offers unique opportunities but also introduces significant challenges.

We propose a four-stage framework for data linkage: (1) understand participant expectations and acceptability; (2) collect and link the data; (3) evaluate properties of the data; and (4) ensure secure and ethical access for research. We highlight the value of participant engagement for maintaining the trust built by LPS, highlight challenges of ensuring quality and well-documented data when collaborating with third parties, and discuss the value of trusted research environments and synthetic data for protecting participant confidentiality.

We address potential biases in collecting and analysing digital footprint data.

While many of the challenges are not unique to digital footprint data (e.g., sample biases), others are (e.g., individual versus household data attribution). We acknowledge that this framework is developed based on the perspectives of the authors, specific to particular cohort studies and data types. Therefore, we welcome responses from others to further refine the dialogue.

In conclusion, while linking digital footprint data into LPS is in early stages, experience of linking data in ALSPAC and Generation Scotland highlight their potential. Future studies will push the community forward by establishing initial frameworks, such as data sharing protocols, ethical guidelines for consent, and standards for data harmonisation. Iteratively refining these frameworks will ensure robust integration, ultimately contributing to the development of ethical, transparent, and effective methods for leveraging digital footprint data in LPS and beyond.

## Footnotes

1 Authors A.B, O.D, L.M, A.S, M.M, and A.S.

2 Authors S.R. and Z.X.

## Data access statement

This work does not involve new data collection. Data for the Avon Longitudinal Study of Parents and Children is described on the study website, (http://www.bristol.ac.uk/alspac/researchers/our-data/); Generation Scotland summary data can be viewed on request by contacting access@generation scotland.org. In both cases, data may be accessed by researchers upon reasonable request.
